# Heat-Induced Structural Changes Affect OVA-Antigen Processing and Reduce Allergic Response in Mouse Model of Food Allergy

**DOI:** 10.1371/journal.pone.0037156

**Published:** 2012-05-21

**Authors:** Jaroslav Golias, Martin Schwarzer, Michael Wallner, Miloslav Kverka, Hana Kozakova, Dagmar Srutkova, Klara Klimesova, Petr Sotkovsky, Lenka Palova-Jelinkova, Fatima Ferreira, Ludmila Tuckova

**Affiliations:** 1 Department of Immunology and Gnotobiology, Institute of Microbiology, v.v.i., Academy of Sciences of the Czech Republic, Prague, Czech Republic; 2 Department of Immunology and Gnotobiology, Institute of Microbiology, v.v.i., Academy of Sciences of the Czech Republic, Novy Hradek, Czech Republic; 3 Christian Doppler Laboratory for Allergy Diagnosis and Therapy, Department of Molecular Biology, University of Salzburg, Salzburg, Austria; Université Paris Descartes, France

## Abstract

**Background and Aims:**

The egg protein ovalbumin (OVA) belongs to six most frequent food allergens. We investigated how thermal processing influences its ability to induce allergic symptoms and immune responses in mouse model of food allergy.

**Methodology/Principal Findings:**

Effect of increased temperature (70°C and 95°C) on OVA secondary structure was characterized by circular dichroism and by the kinetics of pepsin digestion with subsequent HPLC. BALB/c mice were sensitized intraperitoneally and challenged with repeated gavages of OVA or OVA heated to 70°C (h-OVA). Levels of allergen-specific serum antibodies were determined by ELISA (IgA and IgGs) or by β-hexosaminidase release test (IgE). Specific activities of digestive enzymes were determined in brush border membrane vesicles of jejunal enterocytes. Cytokine production and changes in regulatory T cells in mesenteric lymph nodes and spleen were assessed by ELISA and FACS. Heating of OVA to 70°C caused mild irreversible changes in secondary structure compared to boiling to 95°C (b-OVA), but both OVA treatments led to markedly different digestion kinetics and Tregs induction ability in vitro, compared to native OVA. Heating of OVA significantly decreased clinical symptoms (allergic diarrhea) and immune allergic response on the level of IgE, IL-4, IL-5, IL-13. Furthermore, h-OVA induced lower activities of serum mast cell protease-1 and enterocyte brush border membrane alkaline phosphatase as compared to native OVA. On the other hand h-OVA stimulated higher IgG2a in sera and IFN-γ secretion by splenocytes.

**Conclusions:**

Minor irreversible changes in OVA secondary structure caused by thermal processing changes both its digestion and antigenic epitopes formation, which leads to activation of different T cell subpopulations, induces shift towards Th1 response and ultimately reduces its allergenicity.

## Introduction

Food allergy is a serious health concern affecting 6–8% of young children and about 2–4% of adults. Allergies to eggs, milk and peanut are currently the most frequent food allergies and their prevalence, severity and persistence has been increasing during the last decades. Food allergy is considered mainly as an IgE-mediated type I hypersensitivity, characterized by an increased production of IgE antibodies and Th2 cytokines, common markers found both in human disease and in experimental models [1]–[4].

Depending on the route of exposure, dose of allergen and the presence of suitable adjuvant, the immune response can result in either sensitization or oral (mucosal) tolerance induction [4]–[6]. In mouse models of food allergy, oral administration of allergen usually results in oral tolerance induction, but its co-administration with strong mucosal adjuvant such as cholera toxin or with anti acid drugs (increasing gastric pH) could be used for allergic sensitization [7]–[10]. Another reliable and effective approach to overcome the oral tolerance induction is pretreatment of mice by systemic intraperitoneal (*i.p.*) administration of allergen with aluminum hydroxide (alum) as adjuvant followed by repeated intra-gastric treatments. This experimental model mimics a mild form of human allergy with IgE–mediated mast cell degranulation causing increased small intestine permeability [2], [11], [12] with diarrhea as one of the symptoms of anaphylaxis. Histological examination of small intestine reveals changes of epithelium, *e.g.* alteration in number of goblet cells and mucin production and the damage of tips of villi, as well as changes of lamina propria, *e.g.* increased cell infiltration and/or activation [13]–[15].

The mucosa of small intestine is an actively metabolizing, rapidly proliferating, absorptive epithelium with nutritional and homeostatic functions. The activity of brush border enzymes is sensitive marker of intestinal cell differentiation and postnatal development, reflecting both dietary changes and microbial colonization [16]–[18]. Partial and subtotal atrophy of the villous apparatus was shown to correlate with the activity and expression of alkaline phosphatase [19]. Moreover, this enzyme may be also involved in host’s defense against pathological stress-induced damage, such as during inflammation and infection [20].

Egg white contains several allergens such as ovalbumin (OVA), ovomucoid, ovotransferin and lysozyme. Forming approximately 60% of the total egg white protein, OVA is by far the most abundant of them [21]. Like the majority of food allergens OVA is consumed after thermal processing and it has been shown that after heating its molecular structure as well as allergenicity is altered [22], [23]. However, it should be considered that egg allergens are processed at different temperatures (baked, scrambled or soft/hard boiled eggs or even native as whipped egg white) and these processing conditions can have a major impact on the secondary structure, susceptibility to enzymatic digestion in the gastrointestinal tract and allergenicity. Partial decrease of IgE binding after OVA thermal processing suggested that both linear and conformational epitopes participate in the OVA-IgE specific interactions [22]–[24]. Moreover, heating of allergens can lead to their aggregation, which reduces their absorption and transport through epithelial layer and thus decreases their allergenicity [25]. However, the impact of different temperature treatment on the changes in the secondary structure of OVA and on its ability to induce clinical symptoms of food allergy hasn’t been studied in detail.

In the present study we show that heating of hen egg allergen OVA to 70°C has only minor effect on its secondary structure. However, these minor changes lead to different kinetics and occurrence of fragments after digestion. This result in activation of different T cell subpopulations and changes in both cytokine production and specific antibody formation, which leads to significant reduction of egg allergy symptoms.

## Materials and Methods

### Ethics Statement

All animal experiments were approved by the Laboratory Animal Care and Use Committee of the Institute of Microbiology v.v.i., Academy of Sciences of the Czech Republic, approval ID: 94/2006 and 244/2009.

### Animals

Two month-old female BALB/c mice (*H-2b*) (Animal facility of the Institute of Physiology ASCR, Czech Republic) were kept under standard conditions, fed by OVA-free diet and water *ad libitum*.

### Ovalbumin Preparation

For *i.p*. sensitization, OVA (Worthington, Lakewood, NJ, USA) and heated OVA (h-OVA; prepared by exposure of OVA to 70°C for 10 minutes, enabling accurate and reproducible dosing) were dissolved in phosphate-buffer saline (PBS) to a final concentration of 300 µg/ml containing 5 mg/ml of alum adjuvant (Sigma, Steinheim, Germany). For oral administration, OVA and h-OVA were dissolved in PBS to a final concentration of 100 mg/ml. For *in vitro* studies boiled OVA (b-OVA) was prepared by exposure of OVA to 95°C for 10 minutes. EndoGrade® Ovalbumin (Hyglos GmbH, Germany) with endotoxin content <1 EU/mg was used for enzymatic digestion and *in vitro* stimulation.

### Circular Dichroism

Protein secondary structure elements were determined by CD spectroscopy. Spectra were recorded in 5 mM sodium phosphate buffer (pH 7.4) with a JASCO J-815 spectropolarimeter fitted with a PTC-423S Peltier single position cell holder (Jasco, Tokyo, Japan). All spectra are baseline-corrected and presented as mean residue molar ellipticity [Θ]_MRW_ at a given wavelength. Thermal denaturation of proteins was monitored from 20°C to 70°C or from 20°C to 95°C at the fixed wavelength of 222 nm with a temperature slope of 1°C/min. The melting point (Tm) was calculated from the inflection point of the resulting sigmoid curve [26].

### Enzymatic Digestion and HPLC Separation of Ovalbumin Fragments

Peptides of OVA, h-OVA or b-OVA were prepared using pepsin-agarose gel similarly as described previously [27]. Briefly, digestion of proteins was stopped after 20, 40, or 60 minutes by removing the pepsin-agarose gel by centrifugation (10 min; 1500 g) and by neutralization with 1 M NaOH to final pH 7. Digested or undigested proteins were separated using SP 250/10 NUCLEOSIL 300-7 C18 column (Macherey-Nagel, Düren, Germany) on the HPLC system Gold 125NM Solvent Module (Beckman Coulter, Miami, FL, USA). Samples were applied on columns and separated as described previously [27]. For *in vitro* stimulations, digests were dissolved in complete RPMI-1640 (Sigma-Aldrich, St. Louis, MO, USA) to a final concentration of 500 µg/ml.

### Experimental Protocol

Mice were divided into the three groups according to the treatment – OVA, h-OVA and PBS (controls). Mice were sensitized *i.p*., with a two week interval, with 60 µg of either OVA or h-OVA together with 1 mg of alum in a final volume of 200 µl PBS on day 1 and 14. Control mice received only 200 µl PBS containing 1 mg of alum. Two weeks later, the mice were challenged 10 times at 2–3 days intervals by *i.g.* gavages of 15 mg of OVA in a final volume of 150 µl PBS. Diarrhea was assessed visually by monitoring mice for 30 minutes after each *i.g*. exposure. Body weight was recorded before gavage and rectal temperature both before and 30 minutes after each *i.g.* exposure.

### Quantification of OVA-specific Antibodies and Mast Cell Protease-1

Blood samples were collected before the first *i.p.* injection, during the experiment and at sacrifice. Allergen-specific serum IgG1, IgG2a and IgA levels were determined by ELISA [28]. Briefly, 96-well microtiter plates were coated either with OVA, h-OVA or b-OVA (5 µg/ml). Serum samples were diluted 1/10000 for IgG1, 1/100 for IgG2a and 1/10 for IgA. Rat anti-mouse IgG1, IgG2a and IgA antibodies (Abs) (1 µg/ml Pharmingen, San Diego, CA, USA) were applied, followed by peroxidase-conjugated mouse anti-rat IgG Abs (1/1000; Jackson, Immuno Labs., West Grove, PA, USA) for detection. Antibody levels were reported as optical density (OD). As it was shown that allergen-specific IgG interferes with allergen-specific IgE detection [29], allergen-specific IgE levels in sera were quantified by degranulation of rat basophil leukemia (RBL-2H3) cells (originally described by [30], kindly provided by prof. Ursula Wiedermann). RBL-2H3 cells were plated in 96-well tissue culture plates (4×10^4^ cells/per well) and passively sensitized by incubation with mouse sera in a final dilution of 1/90 for 2 hours. After washing, OVA, h-OVA or b-OVA (0.6 µg/ml) were added for 30 min at 37°C to induce degranulation. Supernatants were incubated with 4-methylum-belliferyl-N-acetyl-β-D-glucosaminide (Sigma-Aldrich, St. Louis, MO, USA) for analysis of β-hexosaminidase using a fluorescence microplate reader (λ_ex_:360 nm/λ_em_:465 nm) Infinite M200 (Tecan Group Ltd., Grödig, Austria). Results are reported as percentage of total β-hexosaminidase release from cells after disruption with 1% Triton X-100.

Levels of serum mouse mast cell protease-1 (MMCP-1) enzyme were determined by commercial kit (eBioscience, San Diego, USA) according to manufacturer’s instructions. Sacrifice sera were diluted 1/250 and the MMCP-1 levels are reported as ng/ml.

### Cell Culture and Cytokine Evaluation

Mesenteric lymph nodes (MLN) and spleens were removed at sacrifice. Single-cell suspensions were prepared in RPMI-1640 containing 10% fetal bovine serum (BioClot GmbH, Aidenbach, Germany) and 1% Antibiotic-Antimycotic solution (Sigma-Aldrich). Cells (6×10^5^/well) were cultured in a flat-bottom 96-well plate (TPP, Trasadingen, Switzerland) without any stimuli or in the presence of either OVA or h-OVA (100 µg/well) for 72 hours (37°C, 5% CO_2_). Supernatants were collected and stored at –40°C until analyses. IL-4, IL-5, IL-6, IL-10, IL-13, IL-17, INF-γ and TNF-α were determined by the MILLIPLEX MAP Mouse Cytokine/Chemokine Magnetic Panel (Millipore, Billerica, USA) according to manufacturer’s instructions and analyzed with the Bio-Plex System (Bio-Rad Laboratories, Hercules, USA) with sensitivities <0.3 pg/ml for IL-4, <0.8 pg/ml for IL-5, <2.1 pg/ml for IL-6, <2.6 pg/ml for IL-10, <12.4 pg/ml for IL-13, <0.7 pg/ml for IL-17, <1.1 pg/ml for IFN-γ and <3.1 pg/ml for TNF-α. Values are reported in pg/ml after subtraction of baseline levels of non-stimulated cultures. Values below assay sensitivity were considered non-detectable (n.d.). In order to measure the capacities of OVA, h-OVA and b-OVA and their peptic digests (100 µg/well) to induce Tregs, we cultivated them with naïve mouse splenocytes for 48 hours.

### Flow Cytometry Analysis

Single-cell suspensions of spleens or MLN were stained for regulatory T cells using Foxp3 Staining Buffer Set (eBioscience, San Diego, CA, USA) with fluorochrome labeled anti-mouse monoclonal Abs: CD3e-Fluorescein isothiocyanate (eBioscience; clone 145-2C11), CD4-Qdot® 605 (Invitrogen, clone RM4-5), CD25-Alexa Fluor® 700 (eBioscience; clone PC61.5) and Foxp3-phycoerythrin (eBioscience; clone FJK-16s) according to the manufacturer’s recommendation. Flow cytometric analysis was performed on LSRII (BD Biosciences, San Jose, CA, USA) and data were analyzed using FlowJo software (Tree Star, Ashland, OR, USA).

### Determination of Enterocyte Brush-border Enzyme Activities

Jejunum was removed, washed with cold saline and brush border membrane vesicles (BBMV) were prepared from jejunal scrapings as described by Kessler *et al.* [31]. Protein concentration in BBMV was determined by the method of Lowry *et al.* [32] using bovine serum albumin, fraction V (Serva, Heidelberg, Germany) as standard. The activity of alkaline phosphatase (EC 3.1.3.1), γ-glutamyltranspeptidase (EC 2.3.2.2), dipeptidyl peptidase IV (EC 3.4.14.5), lactase (EC 3.2.1.23/62/108) and sucrase (EC 3.2.1.48/10) were determined as described previously [33]. Enzyme activities were expressed in nkat/mg protein, 1 nkat being the amount of the enzyme that converts 1 nmol of substrate per second under the given conditions.

### Histology and Morphometry

Intestinal tissue sections were fixed immediately in 4% formalin. The fixed tissues were cut and processed using routine methods. Paraffin sections (5 µm) were deparaffinized in xylene, rehydrated through an ethanol gradient to water and stained by hematoxylin-eosin. Villus height was evaluated under the Olympus BX 40 microscope equipped with Photo camera DP 70 using program QuickPhoto Micro 23 program (Olympus, Japan). The mean height of 20–30 villi ± SEM was calculated.

### Statistical Analysis

Differences between multiple experimental groups were evaluated by one-way analysis of variance (ANOVA) with Tukey’s multiple comparison test, and differences between two groups were evaluated using unpaired two-tailed Student’s *t*-test. Data were expressed as the mean ± SEM unless otherwise stated. GraphPad Prism statistical software (version 5.03 GraphPad Software, La Jolla, CA, USA) was used for analyses.

## Results

### The Effect of Thermal Processing on OVA Secondary Structure and Enzymatic Digestion

**Figure 1:**
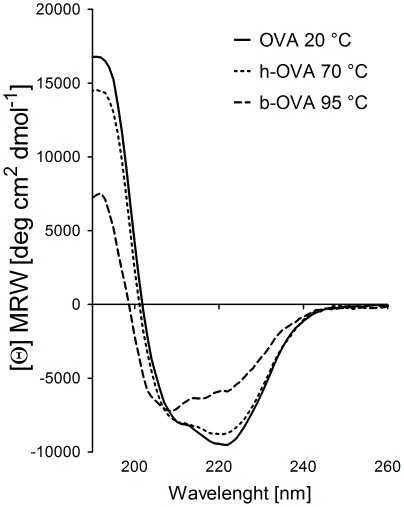
Circular dichroism spectra of native and heated-OVA. Circular dichroism spectra showed only minor irreversible structural changes of hen egg ovalbumin-OVA heated for 10 minutes at 70°C (h-OVA, dotted line) as compared to OVA heated at 95°C (b-OVA, dashed line). Spectra were taken after renaturation at 20°C, native conformation of OVA at 20°C is shown as control (solid line).

**Figure 2:**
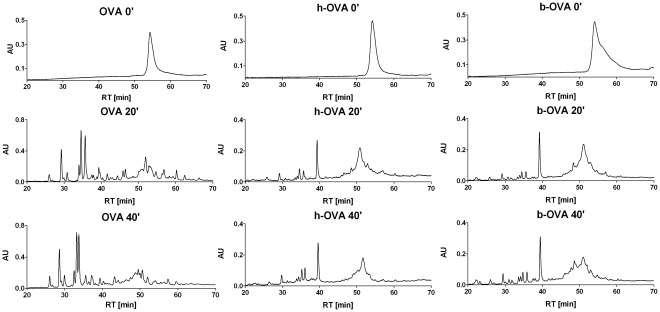
RP-HPLC separation profile of native-OVA and heated-OVA peptic digests. RP-HPLC separation profile monitored at 280 nm corresponds to OVA and OVA heated at 70°C (h)-OVA or boiled at 95°C (b)-OVA undigested (0′) and after 20 (20′) and 40 minutes (40′) of digestion by pepsin. RT – retention time.

Since eggs could be consumed after various kinds of processing, we analyzed the effect of different temperatures on the secondary structure of OVA allergen. Employing the circular dichroism technique, we found that heating to 70°C or 95°C causes irreversible changes in secondary structure of OVA allergen (Fig. 1). The structural changes induced by heating were accompanied by different susceptibility to pepsin digestion. HPLC elution profiles of pepsin-digested OVA, h-OVA or b-OVA were documented after 20 and 40 minutes (Fig. 2). The majority of native OVA was split to fragments after 20 min, while the majority of both forms of heated OVA remained undigested. However, while both h-OVA and b-OVA had similar peptide profiles after 20 or 40 min of digestion, these were both quite different from those of untreated OVA (Fig. 2). The profiles after 40 min of digestion remained almost unchanged after 60 min of digestion (data not shown).

### Experimental Allergic Diarrhea Induced by OVA and Heated-OVA

**Figure 3:**
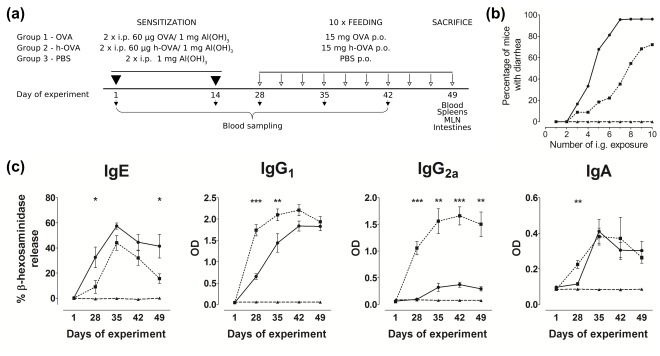
Impact of heating on OVA-induced allergic response. Experimental design (a). Mice were sensitized twice intraperitoneally (*i.p.*) with OVA/Al(OH)_3_, heated OVA (h-OVA)/Al(OH)_3_ or PBS/Al(OH)_3_ alone and subsequently challenged with ten doses of OVA, h-OVA or PBS by intragastric tubing (*i.g*.). Blood samples were taken at indicated time points for antibody analysis. At the end of the experiment, spleens and mesenteric lymph nodes were taken for FACS and cytokine assays, small intestine for histology and enterocyte brush border for enzyme activity analysis. **Occurrence of allergic diarrhea** (**b**). Occurrence of allergic diarrhea in OVA (solid line) or h-OVA (dotted line) challenged mice, data pooled from three independent experiments. PBS controls are shown as dashed line. **The kinetics of specific Abs formation** (**c**). Levels of specific antibodies in sera from mice exposed to OVA (solid line), h-OVA (dotted line) or PBS (dashed line) were detected by ELISA (IgA, IgG1and IgG2a) or by β-hexosaminidase release assay (IgE). Data are represented as mean ± SEM (n = 10 mice/group), representative data from one out of three independent experiments. *P≤0.05, **P≤0.01, ***P≤0.001.

Allergic diarrhea appeared in about 70% of mice already after the 5^th^ *i.g.* dose of OVA, but only in 20% of those fed with h-OVA. After 7 *i.g.* doses, the disease symptoms were found in more than 90% of OVA fed animals, but only in 35% of those fed with h-OVA. At the end of the experiment (10 *i.g.* doses), the diarrhea was found in all mice fed with OVA, but only in 70% of mice fed with h-OVA (Fig. 3a, b). There were small, non-significant differences in body weight and in rectal temperature after each *i.g.* dose of either OVA or h-OVA and PBS control group (data not shown). Morphometry analysis of histological pictures documented shortening of villi in mice treated with either form of OVA, as compared to PBS-treated controls (PBS 190.2±5.1 µm, OVA 157.7±14.0* µm, h-OVA 161.4±6.0** µm).

### OVA and h-OVA Treatment Changes Activity of Brush-border Hydrolases

The brush-border membrane hydrolases are enzymes involved in the final steps of digestion processes. We tested if these enzymes are involved in small intestine homeostasis and could be therefore considered as new markers in food allergy. We determined their activities in the jejunum of OVA-, h-OVA- and PBS-treated mice (Table 1). We found that the specific activity of alkaline phosphatase was significantly higher in mice treated with native OVA but only slightly increased in those exposed to h-OVA, as compared with PBS-treated mice. On the other hand, as compared to PBS-treated controls, both OVA and h-OVA treatments significantly decreased the specific activity of dipeptidyl peptidase IV. We did not observe any significant changes among the three experimental groups in the levels of glutamyl transpeptidase, lactase or sucrase (Table 1).

**Table 1:** Specific activities of enterocyte brush-border enzymes (nkat/mg protein) in jejunum of treated mice.

Enzyme (nkat/mg protein)	OVA	h-OVA	PBS
Alkaline phosphatase	14.26±1.09***	10.08±0.84#	8.27±0.29
GGT	10.79±3.33	8.64±1.59	9.74±2.02
DPP IV	4.61±0.50**	5.39±0.45*	7.39±0.77
Lactase	9.19±0.63	8.37±0.59	9.99±1.61
Sucrase	32.16±8.04	36.00±4.10	27.30±4.24

GGT – gamma-glutamyltranspeptidase, DPP IV – Dipeptidyl peptidase IV. Values are expressed as the mean ± SEM.

* *P*<0.05 ovalbumin-treated group (OVA) *vs.* PBS-treated group.

** *P*<0.01 ovalbumin-treated group (OVA) *vs.* PBS-treated group.

*** *P*<0.001 ovalbumin-treated group (OVA) *vs.* PBS-treated group.

# *P*<0.05 heated-ovalbumin-treated group (h-OVA) *vs.* ovalbumin-treated group (OVA).

### Thermal Processing of OVA Changes the Kinetics of OVA-specific Antibody Responses and the Levels of Serum MMCP-1

To determine the effect of thermal processing of the allergen on the level and specificity of anti-OVA antibodies, the serum levels of IgE, IgG1, IgG2a and IgA against either OVA or h-OVA were determined in the course of the experiment. As shown in Fig. 3c the level of IgE anti-OVA Abs was higher in response to native OVA than to h-OVA. In contrast, OVA-specific IgG2a was significantly higher after h-OVA feeding. The levels of the other two isotypes (IgG1 and IgA) were increased compared to controls but the differences corresponding to the two OVA forms were diminished towards the end of experiment. At the end of the experiment, we characterized the specificity and the degree of cross-reactivity of anti-OVA antibodies using ELISA with OVA, h-OVA or b-OVA bound as an antigen (Fig. S1). The levels of OVA-specific antibodies remained unchanged, when we used h-OVA or OVA as a coating antigen, except for IgG1, which levels were significantly higher, when h-OVA instead of OVA was used. When b-OVA was used as coating antigen, the response of both OVA- and h-OVA treated mice decreased significantly in all measured isotypes.

**Figure 4:**
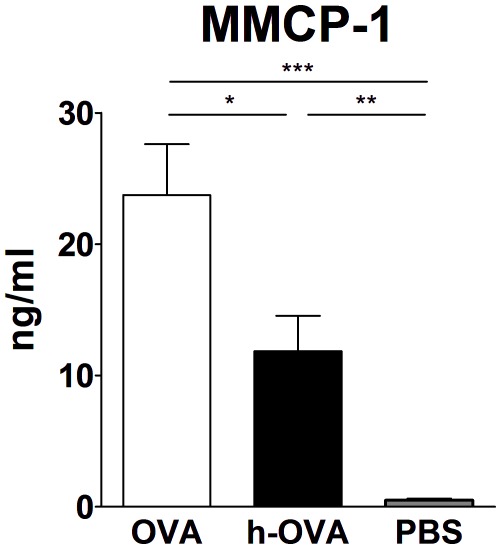
Decreased mast cell protease induction by heated-OVA. Heated OVA (h-OVA, black bar) induced significantly lower amounts of mast cell protease (MMCP-1), the marker of mast cell activation, compared to mice fed with native OVA (white bar). Data are represented as mean ± SEM (n = 10 mice/group), representative data from one out of three independent experiments. *P≤0.05, **P≤0.01, ***P≤0.001.

Increase of allergen specific IgE is essential for mast cell activation and development of allergic diarrhea symptoms. We determined the level of MMCP-1 enzyme as the marker of mast cell activation and degranulation. In this case, the reducing effect of thermal processing was clearly demonstrated; the h-OVA induced only half the level of serum MMCP-1 compared to the native OVA (Fig. 4).

### Ex vivo Cytokine Production by MLN and Splenocytes Induced by OVA Allergens

**Figure 5:**
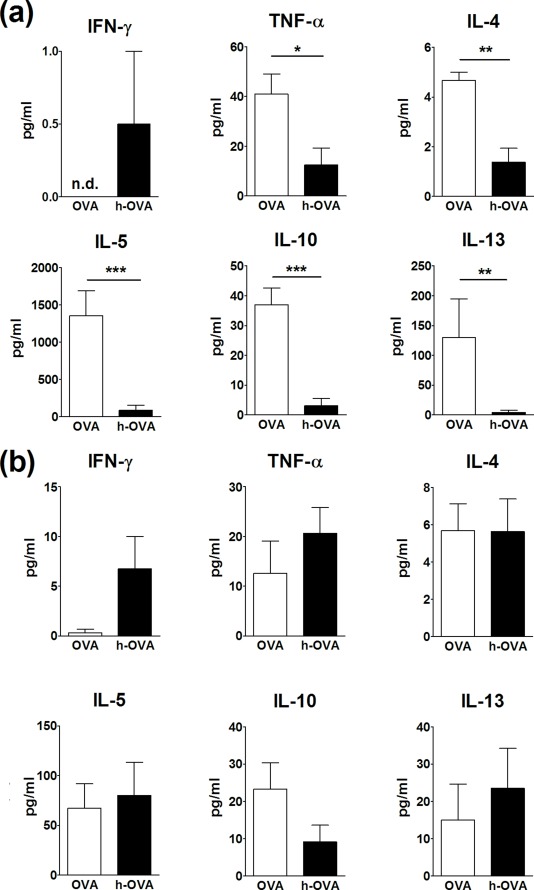
Cytokine production after *in vitro* restimulation with OVA. The cytokine production from mesenteric lymph nodes (a) and splenocytes (b) of BALB/c mice fed with OVA (white bars) or h-OVA (black bars) and stimulated *in vitro* with appropriate allergen. Cytokine levels are expressed after subtraction of base line levels of unstimulated lymph node cells or splenocytes. Data shown are mean values ± SEM (n = 4–7 mice/group), representative data from one out of three independent experiments. *P≤0.05, **P≤0.01, ***P≤0.001, n.d. =  not detectable.

Local and systemic cell responses to OVA and h-OVA were evaluated in all three groups of animals as *in vitro* cytokine production by MLN and splenocytes after exposure to corresponding allergens. Cytokine production from controls (PBS group) was low or not detectable and did not change after exposure to either form of OVA (data not shown). As shown in Fig. 5a, the levels of TNF-α, IL-4, IL-5, IL-10 and IL-13 were higher in culture media obtained from MLN exposed to native OVA. The differences in cytokine secretion were less pronounced in the experiments with splenocytes cultures (Fig. 5b). Only the production of IFN-γ was higher after exposure to h-OVA as compared to OVA. Levels of IL-6 and IL-17 didn’t differ among the groups neither in MLN nor in spleen (data not shown).

### Differentiation of CD4+CD25+Foxp3+ T Cells in OVA and h-OVA Fed BALB/c Mice

**Figure 6:**
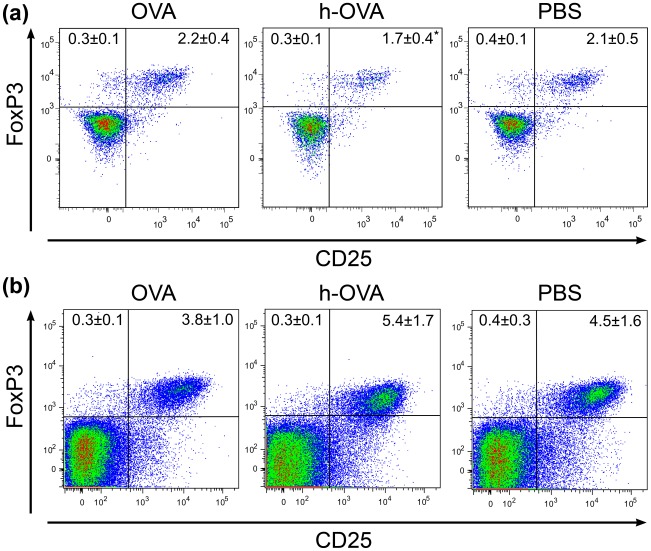
Numbers of Tregs in splenocytes and mesenteric lymph nodes of OVA treated mice. Typical plots depicting numbers of Tregs in mouse splenocytes (a) and mesenteric lymph node (b) in gated CD3+CD4+CD8– T helper cells after feeding with OVA, h-OVA or PBS, respectively. Numbers in upper quadrants shows proportions (mean ± SD) of either CD25–Foxp3+ or CD25+Foxp3+ Th cells out of all cells. Representative data from one out of three independent experiments. *P≤0.05.

Since regulatory T cells (Tregs) are known to be crucial for induction of oral tolerance to protein antigens [34], we analyzed the changes in Tregs in spleen and MLNs of OVA-, h-OVA- and PBS-treated mice at the end of the experiment. In spleen we observed a decrease in Tregs in h-OVA treated mice, as compared to OVA- and PBS-treated mice (Fig. 6). Only a non-significant increase was found in MLNs of h-OVA treated mice.

### Induction of CD4+Foxp3+ T Cells by h-OVA and b-OVA in vitro is Increased After a 20-min Pepsin Digestion

**Figure 7:**
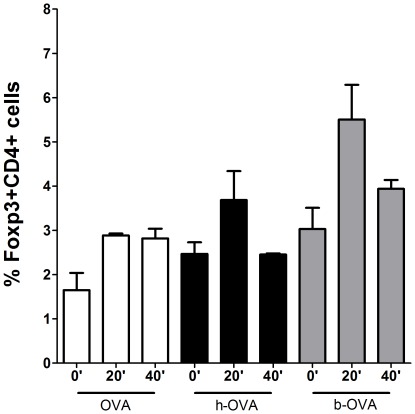
Number of Tregs in spleen cell suspensions co-cultured *in vitro* with OVA digests. The percentage of Tregs in cell suspension isolated from spleens of non-stimulated (naïve) BALB/c mice cultured *in vitro* for 48 hours with undigested (0′) and after 20 (20′) and 40 minutes (40′) peptic digest of OVA (white bars), h-OVA (black bars) or b-OVA (grey bars). The data represent the percentage of CD4+Foxp3+ cells out of all live cells as measured by FACS. Representative data from one out of three independent experiments are shown. Data are represented as mean ± SEM.

To characterize the effect of heating and enzymatic digestion on T cell subpopulations, especially on regulatory T cell differentiation in more detail, splenocytes from naïve (untreated) BALB/c mice were cultured *in vitro* either with OVA, h-OVA or b-OVA as well as with their peptic digests. As shown in Fig. 7, the *in vitro* stimulation of splenocytes with undigested heated proteins led to a slight increase in proportion of CD4+Foxp3+ Treg cells compared to native form of OVA. Interestingly, 20 min peptic digests of heated forms of OVA induced increased proportion of Tregs, but this ability decreased again after 40 min of digestion. In contrast the pepsin digestion did not change the ability of OVA to slightly increase the proportion of Tregs as compared to undigested OVA.

## Discussion

In this study, we showed that small irreversible changes in secondary structure of egg allergen OVA caused by thermal processing significantly affect its digestion by gut enzymes and decrease its allergenicity in the mouse model of food allergy. While both heated and native OVA induced allergic diarrhea in BALB/c mice, the disease symptoms appeared much earlier and with a higher frequency in OVA fed mice than in those fed with h-OVA. As compared to OVA-treated group, the sera of h-OVA-treated mice contained also significantly lower levels of specific IgE and MMCP-1, known markers of mast cell activation and degranulation [35]. It seems that even slight changes in the secondary structure elements have a high impact on the immunological behavior of the allergen. This could be explained by differences either in allergen absorption, which could lead to a decrease in allergen exposure, or in allergen digestion, which leads to production of peptides with different allergenicity and to a partial loss of conformational epitopes and/or exposure of new linear epitopes to immune cells.

The small intestine is noted for its plasticity in response to various dietary changes, which may be reflected in activation of enterocyte brush-border enzymes. Here we demonstrated for the first time that alkaline phosphatase (ALP) can be used as a new marker in food allergy, because its specific activity was significantly increased in OVA-treated group compared to controls. This is in line with the recent findings that ALP has a crucial role in regeneration of enterocytes and that its activity correlates with villous atrophy [19], [36]. We can speculate that the increased level of ALP contributes to restoration of homeostasis in the enterocyte membranes after long-term stimulation with OVA allergens. On the contrary, dipeptidyl peptidase IV (CD26 - that cleaves L-alanine or L-proline residues in the penultimate N-terminal position) was significantly reduced in both OVA- and h-OVA-treated groups, as compared to PBS controls. Interestingly, a decrease in CD26 was found in patients with celiac disease induced by gluten, which belongs to wheat components responsible for food- or wheat-dependent exercise-induced allergy and for occupational asthma [37], [38].

The changes in secondary structure by heating could influence antibody response *in vivo*. Here we report that OVA induced significantly higher levels of OVA-specific IgE and lower levels of IgG2a, as compared to h-OVA. High levels of potentially “blocking” IgG2a (mouse homolog of human IgG4) may compete for allergen [22]. The ability of heat-denatured allergens to induce Th1 associated IgG2a was also shown for other allergens, such as bee venom or birch pollen [39]. However, the effect cannot be generalized, because in a recent study by van der Ventel [15] a higher sensitizing potential was shown for cooked fish proteins. Surprisingly, when we changed the coupling allergen (h-OVA was used for OVA sensitized sera and vice versa) the binding of specific Abs was retained. Moreover, the binding was significantly higher when h-OVA antigen was used for specific IgG1 antibody determination. We assume that this is caused by heating-uncovered linear epitopes (supplementing the loss of the conformational ones), which are then presented after processing by antigen-presenting cells to T and B lymphocytes. On the other hand, when the extensively heated b-OVA was used, we observed a strong drop in the signal in all OVA-specific antibodies, which correlated with observed circular dichroism structural changes, and suggested the importance of structural epitopes in specific antibody formation.

Next, we addressed the question if the differences in OVA and h-OVA-specific antibody responses are also associated with cytokine milieu. On the local level in MLNs, we found a significantly higher production of Th2 cytokines in the OVA-treated mice, accompanied by proinflammatory TNF-α production after an *in vitro* exposure to OVA. Surprisingly, we determined an up-regulation of regulatory cytokine IL-10, which could be a result of a biological feedback aimed at dampening down the local inflammation, similar to chronic experimental colitis [40]. OVA-treatment did not significantly influence cytokine production in splenocytes, except for IFN-γ, which was produced predominantly by h-OVA stimulated splenocytes. The same observation was recently made by van der Ventel [15], who showed an increased IFN-γ production by splenocytes of mice challenged with heated fish extract. Our findings suggest that heating of OVA induces changes in its digestion and processing by immune cells that lead to changes in the local cytokine environment ultimately leading to a shift from Th2- toward Th1-type response, reduction in the level of specific IgE and an increased production of blocking IgG2a antibodies [22]. These data fit well with clinical symptoms observed in allergic subjects in response to heated egg allergens [5], [24].

Moreover, our results support recent data showing that thermal processing interferes with OVA stability [23]. Here, we show that h-OVA and b-OVA are initially (at 20 min) more resistant to proteolysis than native OVA. The difference in degradation kinetics could be explained by partial aggregation of heated forms of OVA, which makes the target structures less accessible for the enzyme. Nevertheless, after 40 min digestion the number of h-OVA and b-OVA fragments was even higher and their spectrum differed from those obtained from OVA. However, the spectra of h-OVA and b-OVA fragmented peptides were similar, differing only in the region corresponding to retention time of 50 min. Surprisingly, when we stimulated splenocytes from naïve mice *in vitro* we found an increase in the percentage of regulatory T cells in response to h-OVA and b-OVA. The capacity of both heated forms of OVA to induce Tregs was increased after 20 min of pepsin digestion and decreased again after 40 min digestion. The prolonged digestion had no effect on Treg inducing capacity of native (heat untreated) OVA digests. These data are supported by recent evidence in experimental mouse model of suppressive effects of some OVA T cell epitope peptides on allergic immune responses via Foxp3+ T cell generation [41].

A direct continuation of the study would be the analysis of intestinal DC subsets and goblet cells [42]–[44] in initial steps of allergen sensitization in our model, which should contribute to understanding how the tolerance or allergic response is achieved. The analysis of the role of enzymes in brush-border membrane of epithelial cells (activated after OVA gavages) will shed light on allergen digestion and immunogenicity of fragments (esp. dipeptidases) and on regeneration of gut epithelium (ALP). Moreover, it would be of great importance to apply this model for verification of hygiene hypothesis using animals kept under conventional and/or germ-free condition and subsequently colonized with various bacterial strains.

In conclusion, we showed that even a mild change in the secondary structure of OVA after thermal processing has far-reaching consequences concerning its antigenic properties. After digestion of h-OVA, fragments with different immunogenic properties are formed leading to the shift from Th2 to Th1-type response as compared to native OVA. Nevertheless, the h-OVA fragments still have the ability to induce allergic symptoms, but these are less pronounced and need longer time to develop.

## Supporting Information

Figure S1 **Cross-reactivity of anti-OVA specific antibodies.** At the end of the experiment we determined the levels of OVA-specific antibodies in OVA and heated (h)-OVA treated mice against OVA, h-OVA (70°C) and boiled (b)-OVA (95°C). The levels were retained for IgE, IgG2a and IgA (a, c, d) when we used OVA as coating antigen for h-OVA-treated mice or h-OVA as coating antigen for OVA treated mice. In case of IgG1 (b) the levels were significantly higher when we used h-OVA as coating antigen for either OVA- or h-OVA- treated mice. When we used b-OVA we observed a significant drop in the signal for all measured antibodies. Representative data from one out of three experiments (n = 8). Repeated measures ANOVA with Tukey’s multiple comparison test was used for analysis of differences between antibody levels of the same sample measured either against OVA, h-OVA and b-OVA antigen. n.s. non-significant, *P≤0.05, **P≤0.01, ***P≤0.001.
(TIF)
