# Correction: Heat-Induced Structural Changes Affect OVA-Antigen Processing and Reduce Allergic Response in Mouse Model of Food Allergy

**DOI:** 10.1371/annotation/53227638-162d-477c-a487-ad856854d823

**Published:** 2012-08-22

**Authors:** Jaroslav Golias, Martin Schwarzer, Michael Wallner, Miloslav Kverka, Hana Kozakova, Dagmar Srutkova, Klara Klimesova, Petr Sotkovsky, Lenka Palova-Jelinkova, Fatima Ferreira, Ludmila Tuckova

In the Funding section, the grant number from the Institutional Research Concept Grant was listed incorrectly. The correct grant number is: RVO: 61388971.

The complete, correct Funding statement is: This work was supported by grants from the Czech Science Foundation (GA310/07/0414, 310/08/H077 and 310/08/H077), http://www.gacr.cz/; [^] Synlab (http://www.synlab.cz), [^] the Academy of Sciences of the Czech Republic (IAA500200801, IAA500200710 and KJB500200904), http://www.gaav.cz/; [^] the Czech Ministry of Education, Youth and Sports (2B06155 and LC07017), http://www.msmt.cz; [^] and by Institutional Research Concept Grant RVO: 61388971, http://www.isvav.cz. [^] The funders had no role in study design, data collection and analysis, decision to publish, or preparation of the manuscript. 

