# Supplementary figures and images for: Heat-Induced Structural Changes Affect OVA-Antigen Processing and Reduce Allergic Response in Mouse Model of Food Allergy
Source: PLoS One. 2012 May 21;7(5):e37156. doi: 10.1371/journal.pone.0037156 (PMC3357411; doi:10.1371/journal.pone.0037156)

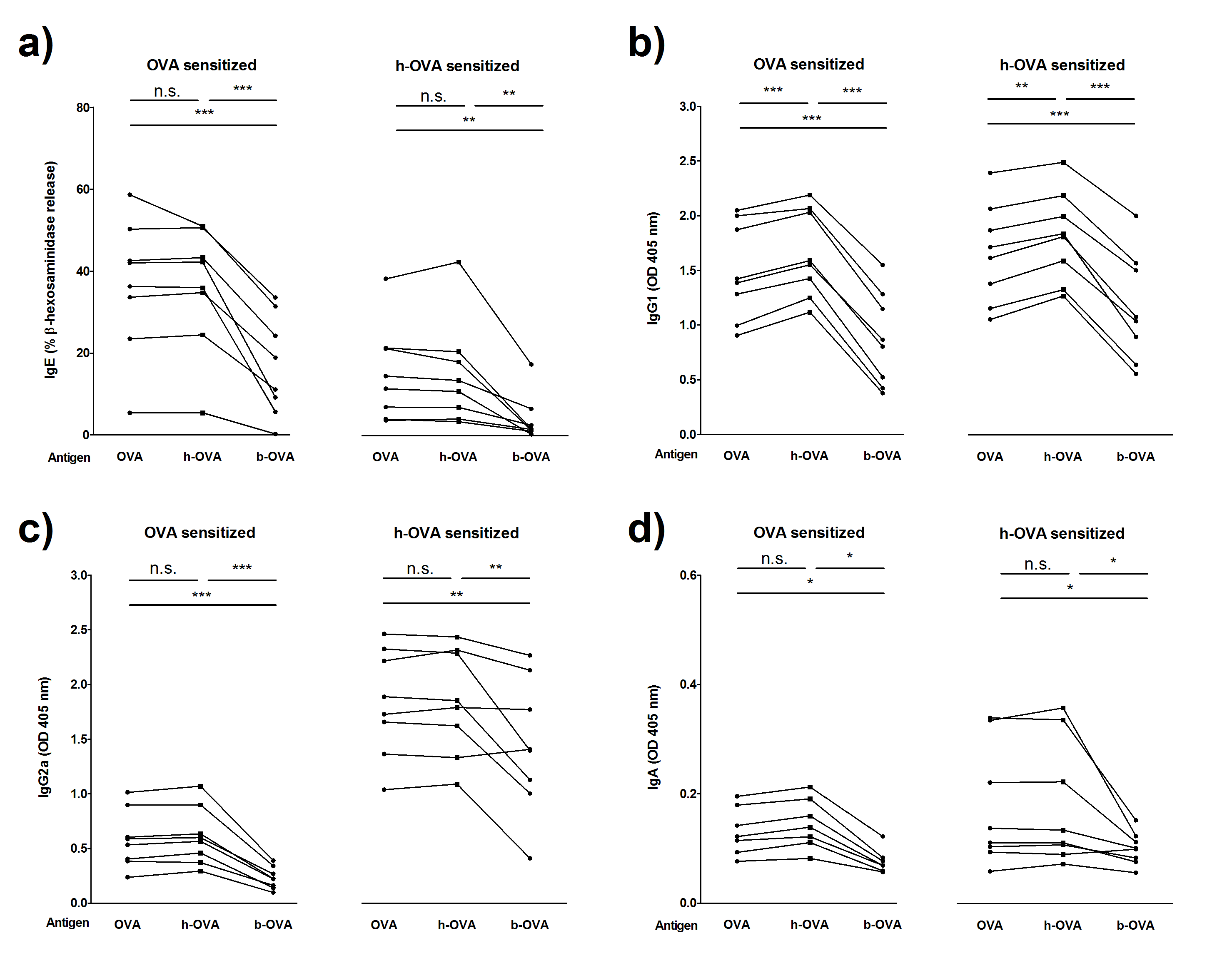

Supplement: Figure S1 — Cross-reactivity of anti-OVA specific antibodies. At the end of the experiment we determined the levels of OVA-specific antibodies in OVA and heated (h)-OVA treated mice against OVA, h-OVA (70°C) and boiled (b)-OVA (95°C). The levels were retained for IgE, IgG2a and IgA (a, c, d) when we used OVA as coating antigen for h-OVA-treated mice or h-OVA as coating antigen for OVA treated mice. In case of IgG1 (b) the levels were significantly higher when we used h-OVA as coating antigen for either OVA- or h-OVA- treated mice. When we used b-OVA we observed a significant drop in the signal for all measured antibodies. Representative data from one out of three experiments (n = 8). Repeated measures ANOVA with Tukey’s multiple comparison test was used for analysis of differences between antibody levels of the same sample measured either against OVA, h-OVA and b-OVA antigen. n.s. non-significant, *P≤0.05, **P≤0.01, ***P≤0.001. (TIF) [file pone.0037156.s001.tif]
